# A Patient With Multiple Sclerosis and Coexisting Moyamoya Disease: Why and How

**DOI:** 10.3389/fneur.2020.516587

**Published:** 2020-10-15

**Authors:** Xiaoli Si, Lingfei Li, Yuanjian Fang, Yaping Yan, Jiali Pu

**Affiliations:** ^1^Department of Neurology, Second Affiliated Hospital, School of Medicine, Zhejiang University, Hangzhou, China; ^2^Department of Neurology, Affiliated Hangzhou First People's Hospital, Zhejiang University School of Medicine, Hangzhou, China; ^3^Department of Neurosurgery, Second Affiliated Hospital, School of Medicine, Zhejiang University, Hangzhou, China

**Keywords:** moyamoya—MM, multiple sclerosis—MS, differential diagnosis, demyelination, immune response

## Abstract

**Introduction:** Multiple sclerosis (MS) and moyamoya (MM) are two separate diseases that rarely coexist. A special case with the two diseases coexisting was reported herein, and previously published articles were reviewed to investigate the clinical manifestations, management, outcomes, and underlying pathogenesis.

**Patient concerns:** A 42-year-old male presented with gradual right limb weakness and slow response for 3 months. However, these symptoms abruptly progressed during his hospital stay.

**Diagnosis:** This patient was diagnosed with coexisting MS and MM finally. The diagnosis of MS was made according to McDonald criteria of multiple lesions and multiple time episodes. Meanwhile, cerebral angiography indicated the diagnosis of MM.

**Interventions:** This patient was treated with methylprednisolone and antiplatelet drug and received bilateral superficial temporal artery bypass surgery for the occulted artery.

**Outcomes:** This patient's right limbs recovered to 4/5-grade muscle strength after 1 month of follow-up after hospital discharge, and his speech function improved after 3 months after hospital discharge.

**Conclusion:** We reported a rare scenario in a patient with the coexistence of MS and MM. We suspect that MS might induce immune response that plays a role in the pathogenesis of MM, while MM might accelerate the demyelination of MS. However, the pathogenesis and therapeutics of MM and MS coexistence need further investigation.

## Introduction

Moyamoya (MM) is a non-inflammatory vascular disease and manifests with occlusions in terminal intracranial internal carotid arteries (ICA), proximal anterior cerebral arteries (ACA), and middle cerebral arteries (MCA) (1). MM is classified into Moyamoya disease and Moyamoya syndrome (2). The etiology and pathogenesis of MM are yet unknown. MM is a progressive vascular pathology that often debuts with a stroke. MM is easily misdiagnosed because it is rare and sometimes is asymptomatic or atypical (3). Multiple sclerosis (MS) is a chronic, progressive, and inflammatory-demyelinating autoimmune central nervous system (CNS) disease. MS has a female preponderance, and its diagnosis relies on clinical symptoms and/or the presence of multiple white matter lesions in magnetic resonance imaging (MRI), demonstrating dissemination in space and time (4), which may share similar clinical features with MM. Unlike MS, there is a definitive treatment for MM (5). Therefore, it is imperative to diagnose MM at the early stage before irreversible cerebral ischemic injury occurs.

The coexistence of MM and MS is rare, and its underlying mechanism is yet unknown. Here we presented a rare case of relapsed stroke which was diagnosed with MS initially; however, it was actually diagnosed with MS and MM simultaneously. Furthermore, we reviewed the previous literatures to summarize the clinical features, treatment effects, and underlying pathogenesis of patients with MS and MM coexistence.

## Case Presentation

**Figure 1:**
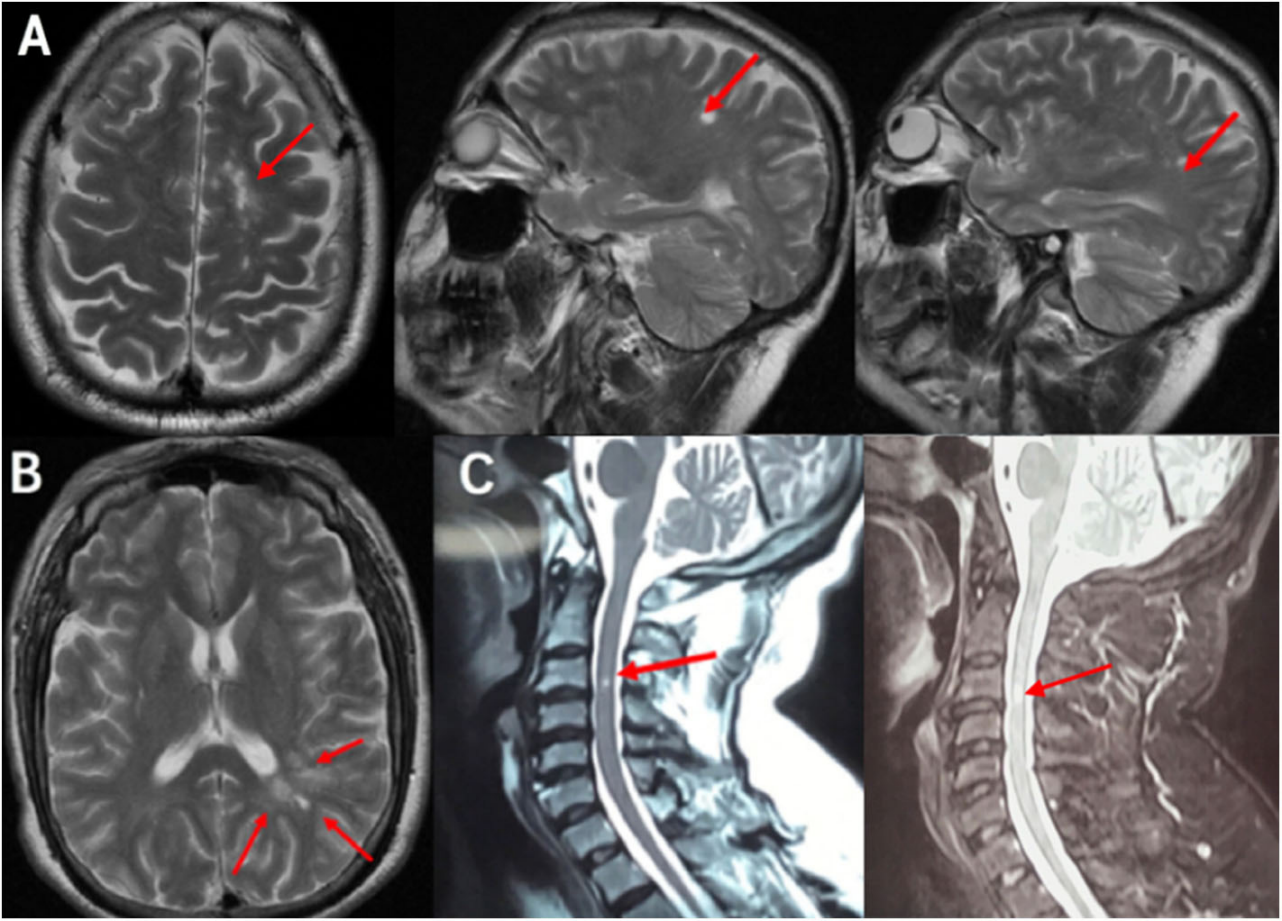
MRI scan demonstrated the DIS: **(A)** Cranial MRI T2 scans revealed three lesions in the cortex and juxtacortical lesions. **(B)** Cranial MRI T2 scans revealed multiple white-matter lesions in the periventricular lesion. **(C)** Cervical MRI scans revealed a cervical 3rd segment with hyperintensity signal in T2 and enhanced T2. Red arrows showed lesions, which was preliminary diagnosed demyelination. MRI, magnetic resonance imaging; DIS, dissemination of lesions in space.

A 42-year-old male primarily complained of gradual onset of bad response and slight inability in the right limbs for 3 months without obvious cause. This patient had no headache and had blurred vision, inarticulate speech, paresthesia, or incontinence. He presented with a history of podagra and mild aortic valve stenosis with insufficiency and denied any history of hypertension, diabetes, hypercholesterolemia, toxic exposure, or head trauma. A family history of similar symptoms was absent. During the pre-hospital phase, his vital signs were stable. This patient underwent a cervical enhanced magnetic resonance imaging (CE-MRI) at a local hospital, which showed a cervical 3rd segment with T2 high signal and with enhancement (Figure 1C). For further diagnosis and treatment, he was hospitalized as an inpatient.

On neurological examination, he was fully alert but revealed a bad response and a declined memory. Mild weakness (4/5 muscle strength) was observed in the right limbs. Bilateral tendon reflex hyperfunction and bilateral Hoffman were positive. No other abnormal findings were detected on physical and neurological examination. Laboratory examination revealed a positive result for hyperhomocysteinemia (homocysteinemia 16.9 μmol/L), hyperlipemia (triglyceride 2.38 mmol/L, high-density lipoprotein 0.88 mmol/L, low-density lipoprotein 3.12 mmol/L), and anti-cardiolipin antibody [anti-β2-glycoprotein I IgA (++), 43.5 RU/ml]. Inflammation markers, thyroid hormone levels, thyroid antibodies, and tumor markers were all unremarkable. Lumbar puncture was carried out after hospitalization to assist the diagnosis. The pressure of cerebrospinal fluid (CSF) was 110 mm H_2_O, and CSF routine was normal. The protein in CSF was 0.401 g/L with Pandy's test weakly positive. The oligoclonal bands (OB) and IgG (0.044 g/L) in CSF were positive. The visual evoked potential (VEP) test was also positive, with abnormal visual pathways in the right and left eyes (P100 wave amplitude reduction). According to cranial MRI, T2-weighted MRI revealed hyperintense signals in the cortex and juxtacortical and periventricular lesions (Figures 1A,B). This patient was primary diagnosed with MS and treated with methylprednisolone (120 mg/d).

**Figure 2:**
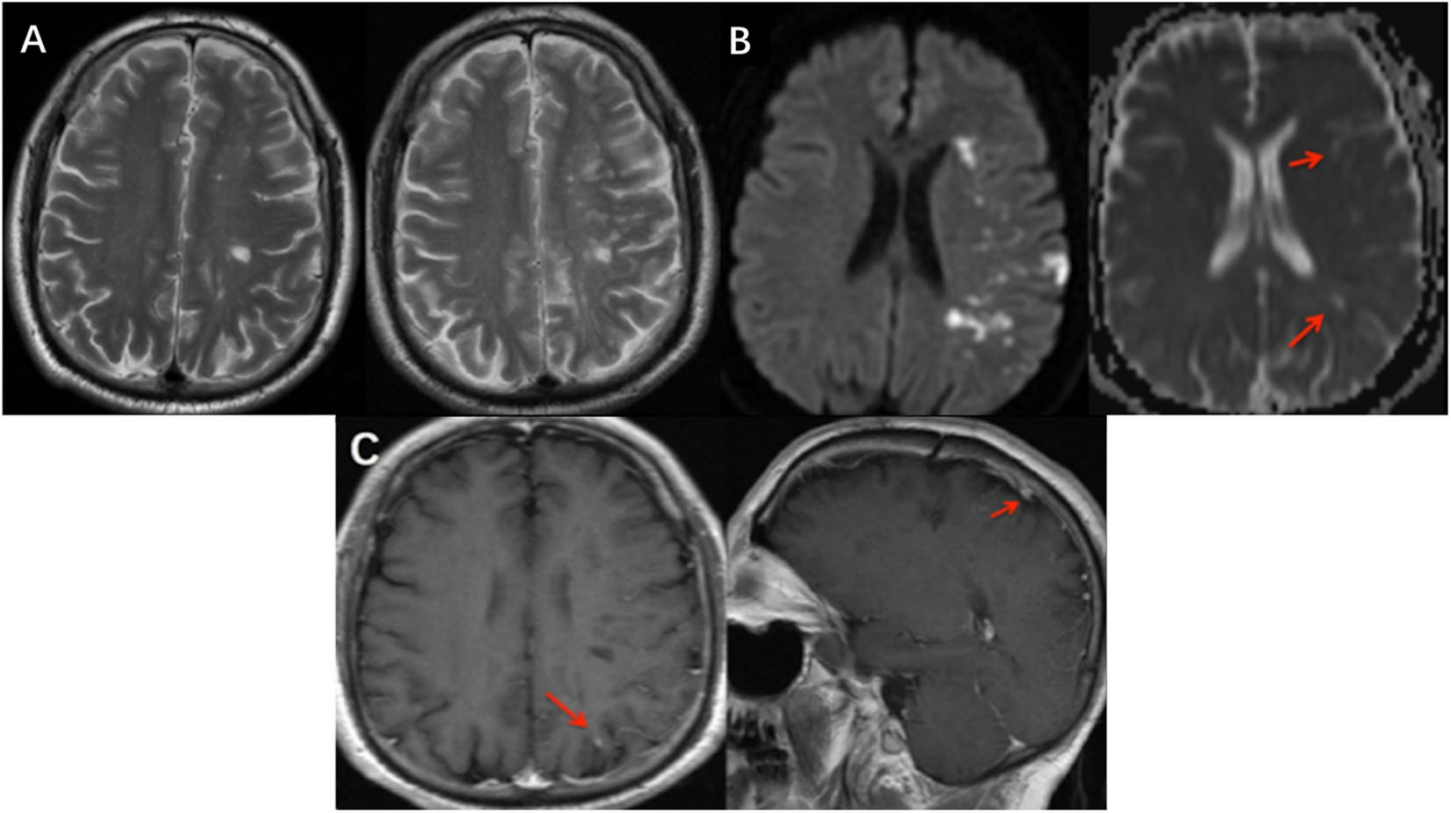
Imaging features at different MRI scans. **(A)** New T2 lesions on EMRI (A-right) compared to the first MRI (A-left) which demonstrated DIT. **(B)** EMRI scan showed hyperintensity in the left frontal–parietal lobe and lateral ventricle nearby in DWI; however, these lesions were both hyperintensity and hypointensity signals in ADC. Red arrows showed hypointensity lesions. **(C)** Cranial EMRI scans revealed that lesion with an enhanced phase signal in the left frontal–parietal lobe (C-left), and also seed enhanced phase lesion in sagittal view (C-right). EMRI, emergency cranial MRI; DIT, dissemination of lesions in time; MRA, magnetic resonance angiography; ACA, anterior cerebral arteries; MCA, middle cerebral arteries; PCA, posterior cerebral artery.

**Figure 3:**
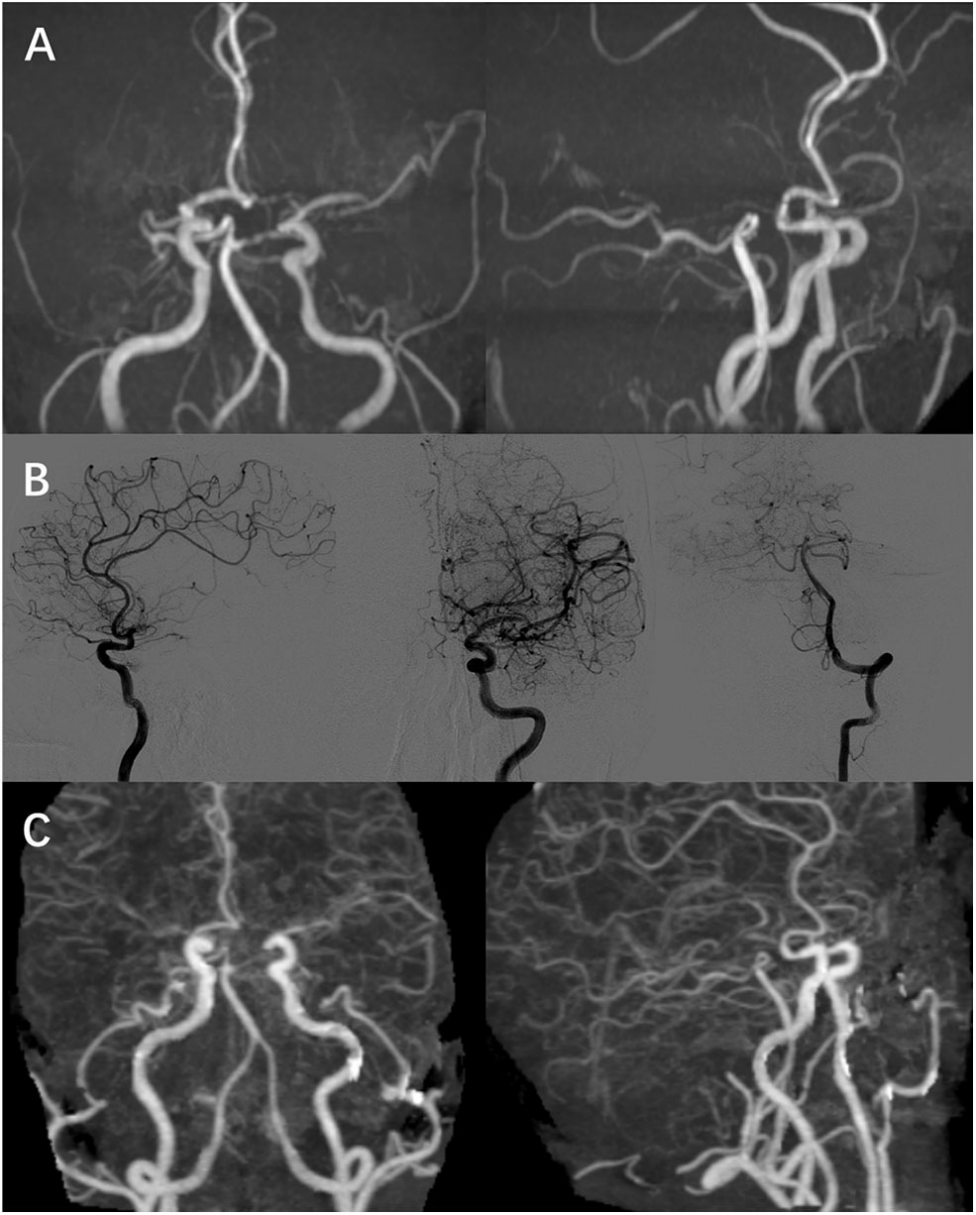
Image result of cerebral vessels. **(A,B)** MRA and DSA showed that right MCA was occulted as well as bilateral terminal ICA, and the left MCA, ACA, and PCA were narrow, which was accompanied with “puff-of-smoke” vessels. **(C)** CTA showed “puff-of-smoke” vessels and meningeal branch neovascularization after the 2-stage bilateral STA bypass surgery. ICA, internal carotid artery; MCA, middle cerebral arteries; ACA, anterior cerebral arteries; CTA, computed tomography angiography.

However, this patient experienced abrupt-onset, aggravating symptoms, presenting with fully alert but worse response and severe motor aphasia; the right side limbs decreased to 2/5-grade muscle strength (National Institutes of Health Stroke Scale/NIHSS 9); and the right-side Babinski sign was positive (his symptoms and neurological examinations were like before when he went to bed last night). This patient did an emergency MRI (EMRI) immediately. EMRI scan showed several new T2 lesions (Figure 2, A-right) compared to the first cranial MRI (Figure 2, A-left), with hyperintensity in the left frontal–parietal lobe and lateral ventricle nearby in diffusion-weighted imaging (DWI); however, these lesions were both hyperintensity or hypointensity signals in apparent diffusion coefficient (ADC) sequence (Figure 2B). We diagnosed him with an acute cerebral infarction. Aspirin (100 mg/d), clopidogrel (75 mg/d), and atorvastatin (20 mg/d) were commenced; meanwhile, the usage of methylprednisolone was ceased. After a few days, this patient did a cranial CE-MRI, which revealed several enhanced signals in white matter of the left frontal–parietal lobe (Figure 2C). Combined with these images, it demonstrated the dissemination of lesions in time (DIT) of demyelination in MS. However, further evaluation with magnetic resonance angiography (MRA) confirmed that his right MCA was occulted, and the left MCA, ACA, and posterior cerebral artery (PCA) were narrow, whose findings were typically seen in MM according to the Research Committee on Spontaneous Occlusion of the Circle of Willis (moyamoya disease) in Japan (6) (Figure 3A).

Three days later, his right limbs recovered to 3/5-grade muscle strength (NIHSS 7). His muscle strength improved to 4/5-grade muscle strength, while trouble was still noted with mild motor aphasia after 1 month of follow-up (NIHSS 3). Further digital subtraction angiography (DSA) was performed after 2 months from hospital discharge. DSA showed that right MCA was occulted as well as bilateral terminal internal carotid artery (ICA), and the left MCA, ACA, and PCA were narrow, which was accompanied with “puff-of-smoke” vessels (Figure 3B). DSA identified the diagnosis of MM, and a neurosurgeon performed bilateral superficial temporal artery (STA) bypass surgery for occulted artery. This patient was reexamined through computed tomography angiography (CTA) after 1 month, which showed “puff-of-smoke” vessels and meningeal branch neovascularization (Figure 3C). Three months after hospital discharge, this patient showed an improvement in both right-limb muscle strength and speech function without relapse.

## Discussion and Conclusion

The coexistence of MM and MS in one patient is extremely rare; only 3 cases have been reported worldwide (5, 7, 8) (Table 1). Here we presented an interesting case with a diagnosis of MS at first but with MS and MM actually coexisting. Collating the history of chronic onset, progressive development, remarkable OB (+), VEP (+), dissemination of lesions in space (DIS), and DIT of demyelination, MS was diagnosed according to McDonald criteria. However, the sudden episode was likely cerebral watershed infarction due to MM, which was proved by MRI and DSA. Therefore, we diagnosed this patient with MM and MS coexistent.

**Table 1:** Neurological presentation and imaging findings of patients presented with MS and MM co-exit.

**Case**	**Country**	**Age /Sex**	**Clinic symptoms**	**White matter hyperintensities in MRI**	**Spinal MRI**	CSF OB^*^	VEP^*^	**Laboratory** **test**	**Angiogram finding**	**Treatment**	**Outcome**
Preziosa et al. (5)	US	44/F^*^	Left hemiparesis	Bilateral subcortical	+	+	–	–	Left ICA^*^ occluded; Right ICA narrow	β-interferon+ Corticosteroids; Bypass surgery^*^	Left hemiparesis with residual; No relapse
Dorfman et al. (7)	US	44/F^*^	Bilateral limbs numbness; diplopia	Bilateral centrum semiovale	–	–	–	Brain biopsy: right frontal lobe demyelinate	Bilateral ICA supraclinoid occluded	Aspirin; Bypass surgery	No relapse
Zaheer and Berger (8)	US	44/M^*^	Right hemiparesis; Impaired vision	Periventricular	–	–	+	ANA^*^(+)	Left ACA^*^ MCA narrow; Left PCA^*^ vasculopathy	β-interferon; Aspirin+ Clopidogrel	No relapse
(this case)	China	42/M^*^	Right hemiparesis; Lags in response	Frontal, parietal, temporal part	+	+	+	anti-β2-glycoprotein I IgA(++)	Right MCA^*^ occluded; Left ACA, MCA, PCA narrow	Corticosteroids; Aspirin+ Clopidogrel; Bypass surgery	No relapse

* *F, female; M, male; CSF, cerebral spinal fluid; OB, oligoclonal bands; VEP, visual evoked potential; ANA, antinuclear antibody; ICA, internal carotid artery; ACA, arteria cerebral artery; MCA, middle cerebral artery; PCA, posterior cerebral artery; Bypass surgery, 2-stage bilateral STA-MCA bypass surgery; STA, superficial temporal artery*.

However, why does this patient have MM and MS? Although genetic factors, bacterial or viral infection, and immune and inflammatory response have been implicated in the development of diseases (9–11), the pathogenesis of MM is still unknown. A current double-hit hypothesis (12) considers a role of immunologic triggers in the context of genetic predisposition (13) of MM. Several studies have demonstrated that MM is an acquired disease and smooth muscle proliferation may result in a mild, chronic inflammatory response with gradual narrowing of intracranial arteries and their branches. Impairment of cerebral circulation may lead to the development of a fragile collateral (14). Reportedly, MM can coexist with immunological disorders such as Sjogren's syndrome (15), advanced acquired immunodeficiency syndrome, and systemic lupus erythematosus (16, 17). In our case, this patient had a history of gout with anti-β2-glycoprotein I IgA (++). Another case was positive in the anti-nuclear antibody test and presented with left PCA vasculopathy, and other immune responses indicated that anti-SS-A and -B antibodies could also be found in MM patients (12–14, 18). MS is known as a chronic, progressive, and inflammatory-demyelinating autoimmune disease of CNS. Studies have suggested that inflammation, neurodegeneration, and regeneration are underlying pathophysiologies of immune response in MS, which can be improved with steroids and immune inhibitors in MS patients (15). Whether there is a direct connection between MM and MS has not been elucidated. We suspected that there might be an interaction between MM and MS and that MS might induce vasculitis which plays a role in the formation of MM, while MM may accelerate the demyelination of MS. The pathogenesis of patients with MM and MS coexistence needs further investigation.

So how can we manage such a patient with MM and MS coexistence for a correct diagnosis and effective therapy? Unlike MS, there are definitive treatments for MM, including surgical bypass interventions and antiplatelet therapy (19). Therefore, it is imperative to diagnose MM at the early stage before irreversible cerebral ischemic injury occurs (20). Although MM is rare, we should also add MM to the list of differential diagnosis of MS, especially in young patients (21, 22). Here is the basis for differential diagnosis. Firstly, MM commonly has paroxysmal onset and recovers within 24 h, whereas MS attacks may progress in a subacute manner after several days (7). Secondly, the clinical presentations of MM include transient ischemic attacks, ischemic stroke, hemorrhagic stroke, seizures, headache, and cognitive impairment (23). These neurologic deficits are related to cerebral hypoperfusion (24). However, symptoms such as blurred vision or spinal sensory or reflex levels indicate the diagnosis of MS. Thirdly, in MM, lesions are especially localized in the frontal and parietal watershed regions and may also affect the deep or superficial gray matter (25), whereas the predilection sites of MS include cortex, subcortex, periventricular, corpus callosum, brainstem, and cerebellum, whose lesions typically are ovoid (26). The key standard for MM diagnosis is intracranial vasculature image (6, 7). Other uncommon causes of stroke, such as cerebral autosomal dominant arteriopathy with subcortical infarcts and leukoencephalopathy, cerebral amyloid angiopathy (27), cerebral venous sinus thrombosis, and dural arteriovenous fistula, should also be added to the list of differential diagnosis of MS (28). However, the treatments of coexistence of MM and MS are controversial. One previous study confirmed that MM might improve or stabilize with a combination treatment of steroids and plasmapheresis (29). In Table 1, we found that patients with MS and MM coexistence, who underwent 2-stage bilateral STA bypass surgery or antiplatelet drug therapy without continual usage of steroids or immune inhibitors, could recover to baseline without relapse, while another study revealed that treatment because of a misdiagnosis as MS bears not only the risk of steroid side effects like Cushing syndrome or diabetes but also the risk for precipitating disabling strokes or hemorrhages resulting from MM pathology (30). In the present case, sudden aggravated symptoms may be caused by cerebral hypoperfusion related to left ICA and MCA. However, the possibility of methylprednisolone treatment for precipitating disabling strokes resulting from MM pathology should not be excluded. Therapeutics of patients with MM and MS coexistence need further investigation.

In conclusion, clinicians should be aware of the clinical and radiological manifestations of vascular syndromes such as MM and appropriately consider these in the differential diagnosis of MS. Moreover, the possibility of a concurrence of MS and MM should not be ignored. The underlying pathophysiology of their interaction and treatment effects necessitates further investigation.

## Data Availability Statement

All datasets generated for this study are included in the article/supplementary material.

## Ethics Statement

The studies involving human participants were reviewed and approved by the ethical approval was obtained from The Second Affiliated Hospital of Zhejiang University School of Medicine's Ethics Committee prior to the study. The patients/participants provided their written informed consent to participate in this study. Written informed consent was obtained from the individual(s) for the publication of any potentially identifiable images or data included in this article.

## Author Contributions

XS and LL: made substantial contributions to design and drafted the manuscript. YF: collected data. YY: revised the manuscript critically for important intellectual content. JP: agreed to be accountable for all aspects of the work in ensuring that questions related to the accuracy and integrity of each part of the work are appropriately investigated and resolved. All authors contributed to the article and approved the submitted version.

## Conflict of Interest

The authors declare that the research was conducted in the absence of any commercial or financial relationships that could be construed as a potential conflict of interest.

## Abbreviations

MM: Moyamoya
MS: multiple sclerosis
MRI: magnetic resonance imaging
CE-MRI: cervical enhanced magnetic resonance imaging
OB: oligoclonal bands
VEP: visual-evoked potential
CNS: central nervous system
CSF: cerebrospinal fluid
DWI: diffusion-weighted imaging
ADC: analog–digital conversion
ICA: internal carotid arteries
DIT: dissemination of lesions in time
MRA: magnetic resonance angiography
ACA: anterior cerebral arteries
MCA: middle cerebral arteries
PCA: posterior cerebral artery
DSA: digital subtraction angiography
STA: superficial temporal artery
DIS: dissemination of lesions in space
CTA: computed tomography angiography.
